# Effective Strategies and a Ten-Point Plan for Cardio-Kidney-Metabolic Health in Croatia: An Expert Opinion

**DOI:** 10.3390/jcm13237028

**Published:** 2024-11-21

**Authors:** Željko Reiner, Bojan Jelaković, Davor Miličić, Marija Bubaš, Ines Balint, Nikolina Bašić Jukić, Valerija Bralić Lang, Vili Beroš, Ivana Brkić Biloš, Silvija Canecki Varžić, Krunoslav Capak, Verica Kralj, Ana Ljubas, Branko Malojčić, Viktor Peršić, Ivana Portolan Pajić, Dario Rahelić, Alen Ružić, Tomislav Sokol, Ana Soldo, Ivan Pećin

**Affiliations:** 1 Division of Metabolic Diseases, University Hospital Center Zagreb, 10000 Zagreb, Croatia; 2 Division of Nephrology, Arterial Hypertension, Dialysis and Transplantation, University Hospital Center Zagreb, 10000 Zagreb, Croatia; jelakovicbojan@gmail.com (B.J.); nina_basic@net.hr (N.B.J.); 3 School of Medicine, University of Zagreb, 10000 Zagreb, Croatia; davormilicic01@gmail.com (D.M.); marija.bubas@miz.hr (M.B.); valerija.bralic.lang@gmail.com (V.B.L.); kcapak@hzjz.hr (K.C.); bmalojcic@gmail.com (B.M.); 4 Department of Cardiovascular Diseases, University Hospital Center Zagreb, 10000 Zagreb, Croatia; 5 Ministry of Health, 10000 Zagreb, Croatia; kabinet@miz.hr (V.B.); ivana.portolanpajic@miz.hr (I.P.P.); 6 Specialist Family Medicine Practice, 10000 Zagreb, Croatia; ines.balint2502@gmail.com; 7 Croatian Institute of Public Health, 10000 Zagreb, Croatia; ivana.brkic@hzjz.hr (I.B.B.); verica.kralj@hzjz.hr (V.K.); 8 Clinical Hospital Centre Osijek, Division of Endocrinology, J. J. Strossmayer University, 31000 Osijek, Croatia; silvija.canecki@gmail.com; 9 UHC Zagreb, 10000 Zagreb, Croatia; ana.ljubas@kbc-zagreb.hr; 10 Department of Neurology, University Hospital Center Zagreb, 10000 Zagreb, Croatia; 11 Medical Rehabilitation of Heart, Lung and Rheumatic Diseases, Thallasotherapia Opatija Special Hospital, 51410 Opatija, Croatia; viktor.persic@medri.uniri.hr; 12 Vuk Vrhovac University Clinic for Diabetes, Endocrinology and Metabolic Diseases, Merkur University Hospital, 10000 Zagreb, Croatia; dario.rahelic@gmail.com; 13 School of Medicine, Catholic University of Croatia, 10000 Zagreb, Croatia; 14 Clinical Hospital Center Rijeka, University of Rijeka, 51000 Rijeka, Croatia; alen.ruzic5@gmail.com; 15 European Parliament, 10000 Zagreb, Croatia; tomislav.sokol@europarl.europa.eu; 16 Croatian Chamber of Pharmacist, 10000 Zagreb, Croatia; ana.soldo@hljk.hr

**Keywords:** blood pressure, arterial hypertension, hypercholesterolemia, LDL cholesterol, cardiovascular disease, stroke, chronic kidney disease, chronic non-communicable diseases, cardio-reno-metabolic health, primary prevention, secondary prevention

## Abstract

Cardiovascular diseases (CVD) are the leading cause of morbidity and mortality worldwide, including in Croatia. Since most patients have multiple disorders and diseases caused largely by the same risk factors, and as it is essential to approach each patient as a person with all disorders, today, we are talking about a new paradigm—cardio-renal-metabolic (CKM) syndrome and cardio-renal-metabolic health, which necessarily includes brain health. Elevated systolic blood pressure, LDL cholesterol, smoking, obesity, diabetes, impaired renal function or chronic kidney disease, which all stem from insufficient physical activity, an unhealthy diet with excessive intake of table salt, and air pollution, are the leading causes of overall morbidity and mortality from CKM diseases, especially mortality from CVD. Experts from various fields key to CKM health have written this document with the aim of integrating it as part of the national plan for the prevention of chronic non-communicable diseases with a focus on CVD, which should become mandatory and be based on the existing guidelines of professional societies.

## 1. Introduction

The National Program for the Prevention and Control of Chronic Non-Communicable Diseases (NCDs) of which cardiovascular disease (CVD) is a key component, follows the *Political Declaration of the Third High-Level Meeting of the [UN] General Assembly on the Prevention and Control of NCDs*, of September 2018, in which Member State governments strongly reaffirmed their commitment to accelerating action on the prevention and control of NCDs, emphasizing the primary role and responsibility for individual action at Member State level, national or local, and collectively at EU level [1]. The World Health Organization’s (WHO) Global Action Plan for the Prevention and Control of NCDs, which aims at “a world free of the avoidable burden of NCDs”, includes nine voluntary global NCD reduction targets to be achieved by 2030 [2]. These nine WHO targets need to be complemented by an increase in health literacy among the general population. All countries should explicitly refer to the WHO’s action plan and commit to supporting all participants in fulfilling commitments guided by the following overarching principles: (1) life-course approach; (2) evidence-based strategies; (3) human rights-based approach; (4) equity-based approach; (5) universal health coverage; (6) empowerment of people and communities; (7) national action, international cooperation and solidarity; (8) multisectoral action. Chronic NCDs, which encompass a range of physical and mental health conditions primarily associated with unhealthy choices and unhealthy lifestyle habits, are the main health issue of today. In Europe, they cause more than 85% of all deaths and 75% of all diseases [3,4]. According to the 2021 report by *Global Burden of Disease* collaborators, published in the Lancet in 2024, the number one risk factor affecting the rates of disability-adjusted life years (DALY) is the environmental risk factor—air pollution, followed by increased systolic blood pressure (BP) (Table 1). For Central Europe and Croatia, which is included in that part of the world for this analysis, systolic BP is ranked first, followed by smoking, elevated glucose levels, obesity, elevated LDL cholesterol and, to the surprise of many, chronic kidney disease (CKD). These are followed by risk factors associated with inadequate consumption of certain foods: excessive drinking of alcohol, insufficient intake of fruit and vegetables, excessive intake of salt, and insufficient consumption of whole grains. Measures that could contribute to effective primary and secondary prevention can be surmised immediately. According to WHO estimates, about 80% of all cardiovascular disease (CVD) and strokes could be prevented if prevention were focused on the main risk factors for NCDs. In addition to mortality, NCDs affect the quality of life. A longer life is not necessarily synonymous with a healthy life and as much as a quarter of life in Europe is spent in poor health. The definition of health, according to the father of preventive medicine Andrija Štampar, reads: “Health is a state of complete physical, mental and social well-being, not just the absence of disease”.

## 2. Current Situation in Croatia

**Figure 1:**
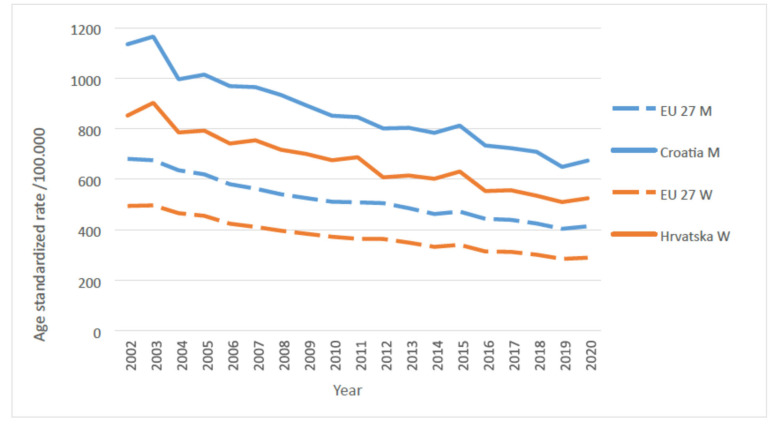
The estimated number of age-standardized deaths from cardiovascular diseases, all age groups, per 100,000 people. Izvor/Source: Eurostat.

The population of the Republic of Croatia is among the “old populations”, with a high share of the population over the age of 65, which has undergone an “epidemiological transition” from the times when infectious diseases were the greatest burden for the population, to the time when NCDs are [5]. In Croatia, CVD has been the leading cause of mortality for decades. In 2022, it was the cause of death for 22,303 people, or 39.1% of all deaths. The analysis by sex shows that CVD is the cause of death of 43.8% of women (12,738) and 34.3% of men (9565). The leading diagnostic subgroups are presented in Table 2. According to the report by the Croatian Bureau of Statistics and the Croatian Institute of Public Health (CIPH), ischemic heart disease is the number one cause of death, accounting for a total of 6925 deaths, while hypertensive disease, which causes 1694 fewer deaths than ischemic heart disease, ranks second, followed by diabetes and cerebrovascular disease. Compared to EU countries, with its standardized mortality rate of 572.8/100,000 Croatia is among the countries with mortality rates above the EU average. The EU27 average is 367.6/100,000, while the rates for EU countries range from 190.4 (France) to 1051.8 (Bulgaria). In the last fifteen years, there has been a downward trend in CVD mortality in Croatia, but CVDs continue to be the leading cause of mortality and morbidity. Compared to 2020, in 2022, there were 664 fewer deaths from ischemic heart disease, 663 fewer deaths from cerebrovascular disease, 230 fewer deaths from DM, but 744 more deaths from hypertensive disease. The age-standardized (according to the 2011 census) CVD mortality rate fell by 29% between 2010 and 2019. However, in the last two years, there has been a stagnation even with a tendency toward increase (Figure 1) [6]. Given the ageing population, socio-economic situation, high prevalence of key risk factors such as arterial hypertension (AH), increased LDL cholesterol in the blood, obesity, DM and smoking, in addition to a high prevalence of CKD and the still excessive intake of salt, it is very certain that the observed trend could become a permanent worsening if comprehensive prevention measures are not taken.

Currently, Croatia has no strategy for the prevention and control of CVD or of most other CNDs. The National CVD Prevention Program was adopted in September 2001; however, it was never operationalized and systematically implemented.

## 3. Most Important Risk Factors for Cardio-Kidney-Metabolic Diseases

Certain risk factors, such as age, gender and genetics cannot be influenced. Proven risk factors for CKM events are AH, dyslipidemias (particularly increased low-density cholesterol (LDL)), obesity, unhealthy diet (excessive intake of salt, insufficient consumption of vegetables and fruits), smoking, elevated blood glucose, insufficient physical activity, excessive alcohol consumption and incipient kidney dysfunction (Table 1) [4].

The American Heart Association has made strongest progress on this issue [7]. These risk factors can be influenced, and they are potentially reversible, which provide a great opportunity for the prevention of CKM diseases. Even small changes in BP and/or LDL cholesterol levels and/or a decrease in salt intake can significantly reduce CKM events and mortality. Arterial hypertension is the most important independent risk factor for CVD and CKD but is also by far the most common NCD. Lipid metabolism disorders, especially elevated LDL cholesterol, are, along with AH, the most important independent risk factor for atherosclerotic CVD (ASCVD). Increased LDL cholesterol is directly and causally associated with coronary heart disease, myocardial infarction, and ischemic stroke. The increased concentration of lipoprotein (a) (Lp (a)) is also a risk factor. An additional problem is the great fear of statins, which strongly affects adherence, but also extremely developed clinical inertia and the tendency among physicians to prescribe statin doses which are too low. We are nowhere close to being satisfied with the results achieved in lowering elevated LDL cholesterol levels in the population, both in primary and in secondary prevention [8]. A particular challenge is familial hypercholesterolemia (FH), as it is the most common hereditary metabolic disease in humans, especially its heterozygous form. FH is often found among persons with ASCVD but is rarely recognized as such and far from being given enough attention. According to recent data, its prevalence in the general population is 1:311, suggesting that only ~1% of potentially affected individuals with FH have been diagnosed [9]. In recent decades, obesity and the body mass index (BMI) have increased significantly worldwide in children, adolescents and adults [3]. The prevalence of overweight and obesity is rising in both developed and developing countries and threatens to halt further reductions in CVD mortality [10]. Obesity, in addition to being a separate NCD, is also a risk factor for the development of other NCDs such as DM type 2, AH, CVD, CKD, and some tumors. The prevalence of DM is on the increase in all age groups in Europe, mainly due to increased prevalence of excessive body mass and obesity, unhealthy diets and physical inactivity. In Europe, 60 million people aged 25 and over suffer from DM [11]. The risk of death in persons with DM is double the risk in those without DM. CKD is an unrecognized global public health problem due to its high prevalence, impact on quality of life, significant increase in overall CV risk, and an enormous burden on the healthcare system [12]. Not even the professional community is sufficiently aware that CKD is a stronger CV risk factor than DM. According to a report published in 2020, CKD is the eighth cause of death in women and the ninth cause of death in men. It is estimated that 8.7% of the adult population in the world suffers from CKD, which should increase public health concerns [13]. By increasing BP at the population level, but also independently, excessive salt intake significantly increases the overall risk of CVD and all fatal sequelae. Excessive salt intake is also associated with a higher risk of CKD, obesity, but also osteoporosis, kidney stones, and some cancers. Therefore, it is not surprising that the WHO has listed excessive intake of salt, or more precisely a 30% reduction in intake, as the fourth among global voluntary targets, and it is also included as one of several “best buy” ways to increase population health. The WHO recommends limiting the intake of salt to no more than 5 g per day. The WHO recommends an average of at least 60 min a day of moderate- to high-intensity activity for children and adolescents, and at least 150 min a week of moderate-intensity aerobic activity or 75 min of high-intensity activity for adults. Regular physical activity has been proven to help in the prevention and treatment of numerous NCDs [13]. Cigarette smoking is responsible for 50% of all avoidable deaths in smokers, half of which are caused by ASCVD [3]. An active smoker has a 50% probability of dying of smoking and will on average lose 10 years of life from smoking. Smokers who quit smoking can reduce their risk of CVD by 39% within five years, but it takes at least five to ten years, and possibly up to 25 years after quitting smoking, to have the same risk of CVD as a person who has never smoked [14]. An additional problem are the new ways to enjoy nicotine by smoking e-cigarettes and heated tobacco. Although these products contain significantly fewer carcinogenic compounds, their less longtime harmful effect on the CV system has not yet been demonstrated, and as they contain nicotine, but also because of their attractiveness, they can create a greater number of young smokers than before; hence, it is necessary to exercise reasonable and strict monitoring of the sale of these products, primarily to young people. The World Heart Federation warns of the harmfulness of these products. Perhaps the greatest benefit of these new products lies in the fact that they are not accompanied by the risk for passive smokers associated with “classic” cigarettes along with potential benefits for smokers who are trying to quit smoking. According to the *Global Burden of Disease* study, fine particles of air pollution (PM 2.5) rank seventh among the risk factors responsible for mortality on the global level, primarily from CVD [4]. Data from 2021 indicate that climate change has increased air pollution-related mortality by 14% [4]. The aim of human biomonitoring is to give the competent authorities a more comprehensive insight into the actual exposure of the population to pollutants, and of vulnerable population groups such as children. In addition to air pollution, noise has been identified as an important CV risk factor. Climate change is strongly linked to air pollution, as large fluctuations and rapid changes in the meteorological situation have been proven to have an adverse effect on overall and CV mortality. It should be noted that annual seasonality is also an important risk factor; hence, these new risk factors, which will unfortunately have an increasing impact in the future, must be considered and included in the national prevention program for all CNDs, especially CVD. Chronic stress, which is an integral part of modern life, has been recognized as an independent risk factor for AH and CVD. Prevention of psychosocial risks, such as chronic stress and sleep disorders, is becoming increasingly important in modern society, where the pace of life is constantly accelerating and pressures on individuals are growing. It is necessary to create an environment that promotes well-being, both in private and professional life. The current situation in Croatia regarding the most important risk factors for cardio-kidney-metabolic health are shown in Box 1A,B.

Box 1 **(A) Most important risk factors for cardio-kidney-metabolic health in Croatia. (B) Most important risk factors for cardio-kidney-metabolic health in Croatia.**

**(A)**

**Hypertension and increased blood pressure**

- In 2022, hypertensive disease (I10–I15) was the second cause of death in Croatia.
- As AH is known to be a key risk factor for ischemic heart disease and the main risk factor for stroke, it can be said without a doubt that AH is the main cause of death in Croatia as well (Table 2)
- An increasing trend regarding the number of deaths from hypertensive disease has been observed in Croatia, with significantly more women than men dying from it. It should be noted that women are almost five times more likely to die from hypertensive disease than from breast cancer. And while the number of deaths from breast cancer is decreasing, the number of deaths from hypertensive disease is increasing.
- The prevalence of AH in Croatia is increasing so that today, according to preliminary results of the national EH-UH 2 study), 50.9% of the adult population has AH. Even though many people with AH are treated (87%), control has been achieved only in 49%.

**Dyslipidemia, obesity, diabetes and chronic kidney disease**

According to preliminary data from the EH-UH 2 study, the prevalence of elevated LDL cholesterol (LDL > 3.0 mmol/L) is over 60%. There is no difference in prevalence between the continental and Mediterranean regions of Croatia, but there is a significant difference between urban and rural areas. Namely, the prevalence of dyslipidemia in rural areas is even higher and exceeds 70%. All this is contributed to by low awareness of the problem of dyslipidemia and an inadequate therapeutic approach. In Croatia, 26.9% of adult women and 27.0% of adult men are living with obesity. Croatia’s obesity prevalence

is higher than the regional average of 25.3% for women and 24.9% for men [15]. The prevalence of DM in 2022 in Croatia was 11.15%, and it ranked third on the list of leading causes of death in 2022, with a 7.8% share in total mortality and an ongoing upward trend in recent decades [16].

     It is estimated that 8.7% of the adult population in the world suffers from CKD, which should increase public health concerns [17]

     Data for Croatia obtained in the national EH-UH 2 study fit into this, and less than 10% of persons with CKD  are aware of having this disease.

(**B**)

**Salt intake, physical activity, smoking**

- According to the results of the EH-UH 1 study, in 2006, the average intake of salt in Croatia was 11.3 g per day (13.3 g in men versus 10.2 g in women) [18].
  - Extraordinary success of the salt reduction program in Croatia was achieved and is shown in Box 2.
- The 2018 European Health Interview Survey (EHIS) conducted in Croatia found that 19.5% of persons aged 15 to 65 meet the WHO recommendations for physical activity (22.7% of male and 17% of female respond-ents) [19].
- Eurobarometer data from 2022 reported that 40% of respondents in Croatia never exercise and 6% exercise regularly (at least 5 times a week).
- Physical activity level in Croatia is not in accordance with the recommendations, either in children and ad-olescents or in adults.
  Despite the major public health problem that physical inactivity in Croatia represents, so far, no strategic documents have been adopted that would provide the preconditions to combat and improve this issue effi-ciently.
- The 2019 European Health Interview Survey (EHIS) found that 22.1% of adults in Croatia are daily smokers, of which 25.6% are men and 19.5% women. Smoking is more common in Croatia than in most EU countries, especially than in more developed and wealthy ones.

**Air pollution and climate changes**

- The WHO has estimated that air pollution in the environment accounts for 0.6% of the top ten risk factors associated with overall mortality in Croatia [20]. According to WHO estimates, about 2% of total deaths in Croatia are related to air pollution [21].
  There is currently no systematic implementation of targeted human biomonitoring in Croatia.
- Preliminary EHUH 2 data analyses revealed a significant difference in systolic and diastolic BP, heart rate and ePWV across seasons, as well significant correlation between all parameters and mean daily 2 m air temperature. There was nonsignificant trend in association between systolic BP and air humidity. An inverse association between systolic BP, diastolic BP and heart rate with air temperature was found confirming sea-sonal fluctuations. Air temperature had an impact on long-term BP variability but also on ePWV which should be considered in clinical research as well as in regular clinical work [22].

## 4. Preventive Measures and Preventive Health Programs

Most deaths related to NCDs, especially those associated with the CKM syndrome, can be avoided by prevention or timely treatment. In the period from 2011 to 2020, a decrease in avoidable mortality rates was observed in both Croatia and EU27. In 2011, 414.75 deaths per 100,000 inhabitants in Croatia could have been avoided by prevention or treatment, and in 2020, 395.15 deaths per 100,000 inhabitants. During this whole period, the rates of avoidable mortality recorded in Croatia were higher than those in EU27. Mortality rates for preventable diseases are consistently higher than mortality rates for treatable conditions [23,24].

**Figure 2:**
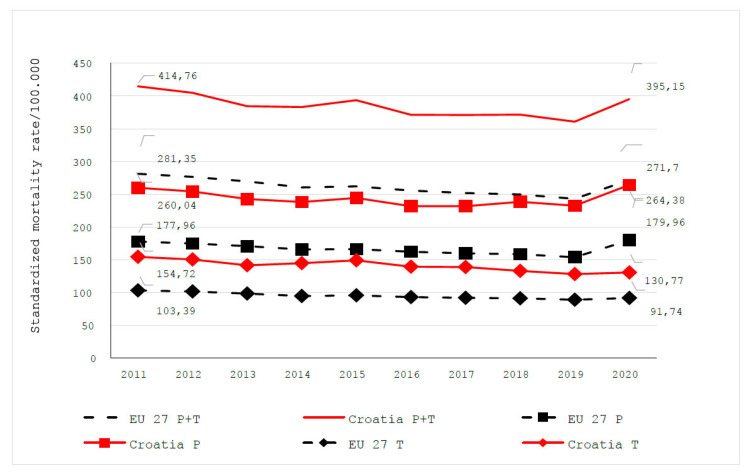
Avoidable mortality (preventable and treatable) in Croatia and European Union (EU27) from 2011 to 2020; P + T = preventable and treatable; P = preventable; T = treatable; Izvor/Source: EUROSTAT.

**Figure 3:**
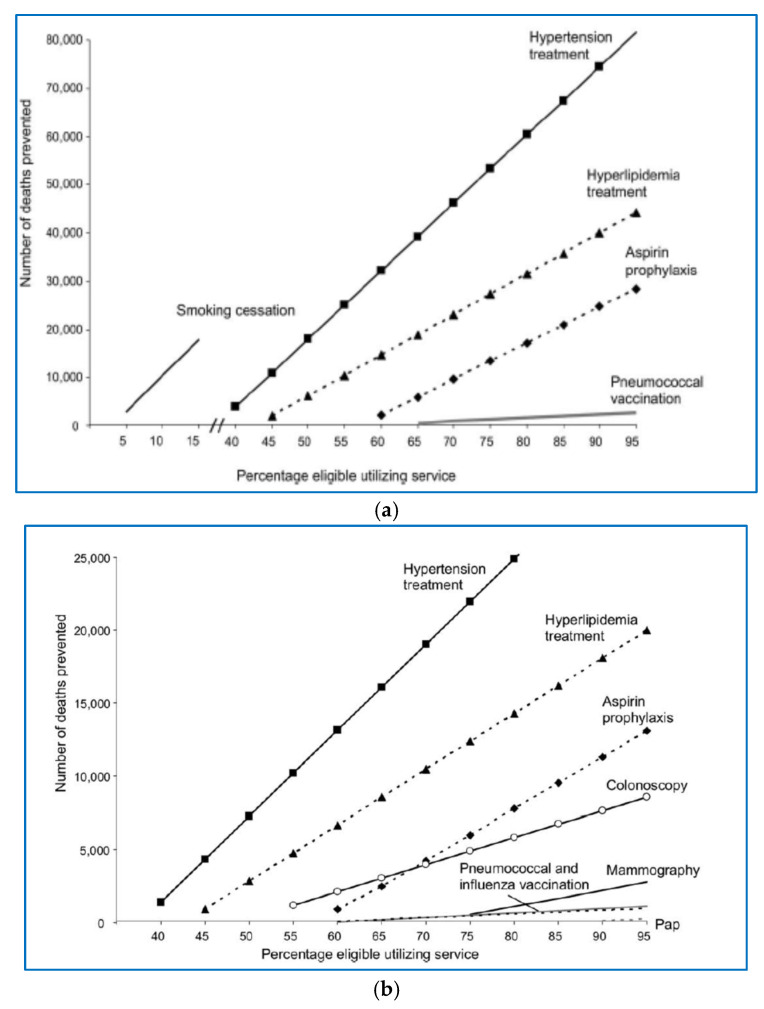
Most effective approaches and measures for saving lives in the general population ((**a**) all-cause model; (**b**) cause-specific model). With permission from Am. J. Prev. Med. [24].

Driven by the question of how the healthcare system can save the most lives, a study was conducted in the United States in which several models were analyzed. The conclusion was that any reduction in the prevalence of AH by 10% results in 14,000 fewer deaths, and any reduction in LDL cholesterol by 10% results in 8000 fewer deaths per year in people over the age of 80. Quitting smoking is associated with an additional 7000 fewer deaths per year (Figure 2). Optimal use of preventive interventions can lead to a total of 50,000 to 100,000 fewer deaths per year in people over 80 years of age, and to 25,000 to 40,000 fewer deaths per year in people aged 65 [24] (Figure 3).

A recently published international study analyzed the effect on overall mortality in the general population of three, as described by the authors, highly effective and feasible interventions: (i) an increase in control of treated individuals with AH of up to 70%; (ii) a 30% reduction in table salt intake; (iii) removal of trans-saturated fats from the diet [25]. This paper analyzes recent meta-analyses and epidemiological studies. The combined effect of these three interventions can reduce the number of deaths by 94.3 million over 25 years, with the effect of improved AH control up to 70% (or lowering systolic BP by 15 mmHg) being associated with 39.4 million, and a reduction in table salt intake by 30% with 40 million fewer deaths. Croatia should be proud of the achievements obtained during the 17-year-long activities for salt reduction (Box 2).

Box 2 **Croatian action on salt and health—a positive example.**

- The Croatian Society for Hypertension, Croatian Society for Atherosclerosis, Croatian Hypertension League, Croatian Institute of Public Health, Croatian Agency for Agriculture and Food, together with other partners organized very extensive and permanent public health activities, aimed at raising awareness of the harm-fulness of excessive consumption of salt and the importance of reducing its intake, and also included negoti-ations with the food industry, where great success was achieved with the largest meat industry PIK Vrbovec, which reduced the content of table salt in all its products by 25% [26].
  - The best example of the multisectoral approach in the prevention and management of NCDs is cooper-ation with the Ministry of Agriculture, which has adopted several ordinances for a gradual but signifi-cant reduction in the content of salt in bread and bakery products, which, according to the current recommendation, should not exceed 1.3%.
  - Following these activities, it was observed that the content of salt in bread and bakery products was 22% lower on average, which shows that most of the bakery industry adheres to the received instruc-tions.
- After activities that lasted over 15 years, and according to the results of the EH-UH 2 study, the intake of salt in Croatia was reduced by an average of 14% and currently amounts to 10 g per day (11.4 g in men and 9.2 g in women), which is primarily a reflection of good cooperation with the food industry [27].
  - The reduced intake of salt was also reflected in lower BP values at the population level, by an average of 3.5/1.9 mmHg.
  - The trend of decreasing CV mortality in Croatia can certainly be largely attributed to the reduction in excessive intake of salt in Croatia.
  - Achievements to date should set an example for all other activities being planned. The path outlined should be continued and strengthened with the additional involvement of the Ministry of Health.

### Programs and Movements to Reduce the Burden of Cardio-Kidney-Metabolic Disease

The EU for Health 2021–2027—A vision for a healthier European Union (EU4Health Program) is a program of action in the field of healthcare from 2021 to 2027, and Regulation 2021/522 of the European Parliament and of the Council of 24 March 2021 places great emphasis on prevention and the promotion of health. The program also aims to reduce mortality from non-communicable diseases by 30% by 2030 [28]. We also think that it is necessary to take the next step and, in a similar vein as with the Europe’s Beating Cancer Plan, create an EU strategy for tackling CVDs, with clearly defined objectives, criteria, benchmarks and strong funding [29].

In the prevention and control of NCDs, and especially CVD, it is necessary to encourage cooperation between all public administration bodies, the local community and all entities with the aim of reducing the burden of disease by developing participatory, cross-sectoral and multilevel cooperation mechanisms that extend from the local to the global level. This requires the involvement of all sectors and entities outside the government: agencies, professional societies and associations, non-governmental organizations, the private sector, and academia. In Croatia, there are several professional societies and associations active in the field of NCD prevention. For many years, the movement of healthy cities and counties and healthy schools started by the CIPH has been developed. Importantly, there are few programs where several NGOs, professional societies, league and institutions came together for joint implementation (Box 3 and Box 4). All these measures and activities together contribute to creating a healthier and more active community, reducing the risk of NCDs, and improving the overall quality of life. Unfortunately, there are still no systematic prevention programs in Croatia, and the system of monitoring risk factors for NCDs, and thus for CVD, is the least developed. So far, only a pilot project of preventive health checkups has been carried out in Croatia in cooperation with the Ministry of Health, health centers, the CIPH and the Croatian Health Insurance Fund. It is a plan to start with the implementation of the National Program of Preventive Health Checkups.

Box 3 **Strategies, projects, actions and movement to reduce the burden of cardio-kidney-metabolic diseases in Croatia by increasing health literacy and by organizing examinations of the population in remote areas.**

- Our example is the Croatian Hypertension League, which includes all relevant professional medical societies related to CKM health, as well as the societies and chambers of nurses and pharmacists, and societies, asso-ciations and boards of nutritionists, kinesiologists, psychologists, academia (Croatian Academy of Science and Arts, schools of medicine and kinesiology, faculties, medical high schools, polytechnics), together with citizens’ associations (Healthy Day Association), patients’ associations (Association of Dyslipidemia Pa-tients), the media (Večernji list), and food industry (PIK Vrbovec).
- Through joint synergy, several years ago a campaign named Hunting the Silent Killer was launched, aiming to increase health literacy through various channels among the general population.
- In addition, a series of public health campaigns were organized during which physical examinations of the general population was conducted. The Croatian Hypertension League team goes out into the field mainly in remote areas of Croatia where health care is underdeveloped, unavailable or insufficient. More than a hun-dred people are examined (and educated) per day. The obtained results are quickly available to local family physicians and can be used in everyday work, and in case additional and supplementary workup or inter-ventions are required, experts involved in the work of the Croatian Hypertension League provide rapid ad-ditional care. The data collected in the campaign are used for scientific research which becomes the basis for future planning that can benefit the Ministry of Health.
- This campaign additionally raises health literacy among healthcare professionals involved in it and is espe-cially important for medical students and high school students at medical schools who are an integral part of the team.
- The literacy of healthcare professionals is further increased through the special educational platform of the Croatian Hypertension League organized with their digital partner d8solutions, HealthMed, edited by lead-ing experts in hypertension, cardiology, nephrology, endocrinology, metabolic diseases, neurology and all other professions important for CKM health. Before being uploaded to the website, all materials go through several levels of verification so that this educational platform can be considered safe, but also exemplary.
- Excellent examples of raising health literacy and continuing the education of physicians are the platforms of the Croatian Cardiac Society and the Croatian Heart House Foundation, Guardians of the Heart, and the educative platform Nefro.hr. led by the Croatian Renal Association.
- For several years, the Croatian Heart House has been carrying out various activities to raise awareness of CV diseases, such as the “Resuscitate me” campaign.
- The Croatian Hypertension League, Croatian Society for Hypertension and Croatian Society for Atheroscle-rosis launched a major program in 2023 to raise health literacy among the general population, patients and healthcare professionals on the two main risk factors for CVD—AH and dyslipidemia, under the names 70/26 and Do you know your number? The goal of the first arm of this program (70/26) is to achieve control in 70% of treated individuals with AH by 2026, and the goal of the second (Do you know your number?) is to significantly reduce the prevalence of hypercholesterolemia and increase control of treated individuals [29]. Education will be provided through various channels as already mentioned, and it should also be noted that the effect on the shifts will be determined by exact measurements. Namely, the baseline data for both im-portant factors of CV, but also cerebrovascular and renal disease, exist from the EH-UH 2 study. At the end of the program, i.e., after two years of education implementation, the same measurement methodology, la-boratory analysis and questionnaires used in the EH-UH 2 project will be repeated.
- The School of Communication organized by the Croatian Hypertension League is aiming to increase the skills of communication between physicians, but also between physicians and patients, and among all healthcare workers. Good communication is a crucial step in increasing awareness, but also in decreasing clinical inertia.
- Another outstanding example of multisectoral cooperation is screening for FH, which in Croatia, at the ini-tiative of the Croatian Cardiac Society and the Croatian Society for Atherosclerosis, began in February 2023 as a special program of the Ministry of Health and the Croatian Institute of Public Health. In Croatia, 2023 saw the beginning of the implementation of the National FH Screening Program with reverse cascade screening that includes the screening of children as part of the physical examination of all children enrolling in the first grade of primary school by determining total blood cholesterol, and then of parents and other close blood relatives. In its White Paper published in June 2021, the World Heart Federation (WHF) included this model as a recommended example of a comprehensive universal national screening program.
- Croatian Cardiac Society and Croatian Society for Atherosclerosis have started with an educative campaign “Cholesterol—good, bad, and inherited”.

Box 4 **Strategies, projects, actions and movement to reduce the burden of cardio-kidney-metabolic health in Croatia by increasing physical activity.**

- The “Living Healthy” National Program continuously promotes physical activity to prevent overweight and obesity. In addition to educating teachers and students about the importance of physical activity in preserv-ing health, physical activity is encouraged through two additional programs: a daily 10-minute exercise and the so-called Polygons for Physical Activity of School-Aged Children. The importance and innovativeness of the Polygons have been recognized by the European Commission, which awarded them as one of the 16 best models of good practice in the field of healthcare, education and sports.
- The Croatian Hypertension League and its two active professional societies, the Croatian Society for Hyper-tension and Croatian Society for Atherosclerosis, together with Run Croatia, have launched several initia-tives aimed at increasing physical activity in the general population as one of the key preventive measures for all NCDs. These activities will take place by organizing races (running, walking, cycling…) for all ages when holding public health actions organized by the Croatian Hypertension League throughout the year.
- Another way to encourage and motivate our people of all ages to walk, run, ride a bike, etc., will take place digitally through a program/application called Go Coin that delivers a reward for movement into the hands of the active person, i.e., in their smartphone. The glook.me powered by the Run Croatia application, which has been further developed with the Croatian Hypertension League, encourages activity and a healthy life-style by rewarding movement. All rewards are adapted to age and needs, and at the same time further en-courage a healthy lifestyle. This application and this way of promoting a healthy lifestyle as a key measure of primary prevention is also one of the successful examples of multisectoral cooperation by the Croatian Hy-pertension League where professional societies (Croatian Society for Atherosclerosis and Croatian Society for Hypertension) have involved and connected several important industries.

So far, three national health surveys have been conducted, in 1997, 2003 and 2008. By joining the EU, Croatia joined the European EHIS survey, which provides internationally comparable results on population health, including NCDs. In Croatia, two national studies have been conducted, EH-UH 1 and EH-UH 2, which determined all the main risk factors for AH, CVD, cerebrovascular disease, CKD, DM, obesity and others, representing a foundation for further evidence-based planning of activities and monitoring of the impact of the activities and measures taken, which is already being carried out and was mentioned earlier in the example of reducing excessive intake of salt.

The CIPH and Croatian Health Insurance Fund developed a system of preventive panels aimed at monitoring information on risk factors for individuals covered by basic health insurance at the level of primary healthcare. The collection of this information has started and will allow for monitoring of the underlying risk factors among the population, while also serving as a tool to focus preventive activities at primary healthcare level. Also, a hospital information system has been developed to enable the study of morbidity and mortality by applying the geographic information system methodology. The inclusion of environmental indicators in these systems will allow us to study the impact of the environment on NCDs.

All these examples show that there is not only a great desire and need among professionals to implement preventive programs, but also that they are feasible. It is therefore necessary to design and organize permanent, systematic and binding prevention programs as part of the national programs for all NCDs, and in particular CVD, building on the experience of these positive examples.

## 5. A Ten-Point Plan for Cardio-Kidney-Metabolic Health and for Reducing Cardiovascular-Disease Morbidity and Mortality in Croatia

The greatest benefit and greatest success in terms of reducing CV morbidity and mortality in Croatia can be achieved by systematic organization of primordial and primary prevention programs that can change poor lifestyle habits by raising awareness and health literacy, and society as a whole must take decisive concrete steps to improve public healthcare with a multisectoral approach (e.g., regulated cooperation with the food industry to further reduce excessive salt intake, changes in school and university curricula, introduction of more physical education classes, mandatory primary physical examinations, etc.). The next thing that can most effectively and economically increase CV health, but also reduce the burden of all NCDs, is to educate patients about the importance of regular medication (increasing adherence), and to continuously train physicians to reduce clinical inertia significantly. In parallel with the digitalization which has become an integral part of modern life, it is necessary to include pharmacists who, with their knowledge and will, can further educate patients and the general population about the necessity of changing poor lifestyle habits, and help significantly in increasing adherence. Besides these basic and key steps, in this publication, we have summarized ten points that should be a part of the future national NCD-CKMs prevention plan, all with the aim of raising the level of care for CKM patients (Table 3). In addition to what is mentioned in these ten points, it is important and necessary to consider cognitive, psychological and behavioral wellbeing and include brain health together with mental health in the program. Finally, environmental risk factors—air pollution and noise—must have a clear place in the program, where certain measures must be proposed that will also have to be tackled through a multisectoral approach (e.g., limiting the driving speed of vehicles in populated areas to 40 km/h, installing sound barriers, etc.). Unfortunately, we cannot influence climate change and seasonality too much, but instructions to patients and to healthcare professionals on how to deal with these situations must be an integral part of the program.

The entire program must feature a strong emphasis on increasing adherence and reducing clinical inertia, because without this, it would be impossible to achieve target values and properly implement secondary or tertiary prevention. One of the important elements is to significantly improve communication between physicians, between doctors and nurses, physicians and pharmacists, and of course between physicians and patients. In the current curricula, this is still deficient and must be improved by employing a multisectoral approach. Physicians are aware of how important this is as evidenced by the excellent response and interest shown in the School of Communication organized by the Croatian Hypertension League.

**Limitations and Strengths**

## 6. Conclusions

Cardiovascular diseases are the leading cause of morbidity and mortality worldwide, Croatia being no exception, and AH is the most important risk factor followed by dyslipidemia. Given the ageing population, pervasive globalization and urbanization, socio-economic situation and high prevalence of risk factors, an increasing burden can be expected not only of CVD but of all CKM diseases if comprehensive prevention measures are not taken. However, every individual should strive to preserve their health by maintaining a healthy lifestyle and, if necessary, taking the prescribed medications. To reduce the incidence of CKM, it is necessary to influence the reduction and/or treatment of the risk factors or causes of these diseases, namely AH, total and LDL cholesterol, DM, CKD, obesity, smoking and inadequate diet with an excessive salt intake and low potassium intake, sedentary lifestyle with insufficient physical activity, stress, air pollution and noise. The approach must be holistic because only in this way can we preserve and improve the CKM health of the population. In order to further draw attention to this key health issue and reduce morbidity and mortality from CVD and all CKM diseases in Croatia, a group of experts from various fields has prepared this document as an introduction to the preparation of a comprehensive national plan for the prevention of NCDs with an emphasis on CVD, i.e., on cardio-kidney-metabolic health, which will have to be concrete, viable, binding and based on the existing guidelines of professional societies.

## Figures and Tables

**Table 1:** Disability-adjusted life years (DALYs) and risk factors relevant to Croatia.

Position by Importance in GBD Report for the World	Position by Importance Among the First 10 Risk Factors According to GBD Report for Central Europe, Incl. Croatia	Risk Factor	Percentage of Total DALY for 2021	Percentage Change 2000–2021 (Age-Standardized)	Percentage Change for Central Europe
1	1	Elevated systolic BP	7.8 (6.4 to 9.2)	−24.3 (−28.4 to −20.0)	−3.2 to −2.2
3	2	Smoking	5.8 (4.7 to 19.9)	−34.8 (−39.2 to −29.7)	−2.2 to −1.6
5	4	Increased FBG	5.4 (4.8 to 6.0)	7.9 (3.3 to 12.9)	−0.6 to 0
6	3	Increased BMI	4.5 (1.9 to 6.8)	15.7 (9.9 to 21.7)	−0,6 to 0
7	5	Elevated LDL cholesterol	3.0 (1.9 to 4.2)	−26.1 (−29.6 to −22.4)	−3.2 to −2.2
8	8	Kidney dysfunction/CKD	3.0 (2.6 to 3.4)	−12.4 (−16.5 to −7−9)	−2.2 to −1.6
10	6	Excessive alcohol consumption	2.5 (2.1 to 3.1)	−25.8 (−32.0 to −20.4)	−1.6 to 0.6
12		Insufficient fruit consumption	1.5 (0.6 to 2.3)	−26.6 (−30.9 to −20.5)	
14	7	Excessive salt intake	1.4 (0.3 to 3.2)	−26.8 (−40.9 to −19.1)	−3.2 to −2.2
15		Insufficient intake of whole grains	1.4 (0.6 to 2.1)	−23.3 (−26.9 to −19.5)	−3.2 to −2.2
24	9	Insufficient vegetable intake	0.7 (0.4 to 1.0)	−28.5 (−33.4 to −21.3)	

FBG = fasting blood glucose, BP = blood pressure; CKD = chronic kidney disease. Adapted from: GBD 2021 Forecasting Collaborators [4].

**Table 2:** Ten most common causes of death in Croatia, according to the 2022 report.

Rank	ICD-10 Code	Diagnosis	Number	%
1	I20-I25	Ischemic heart diseases	6925	12.2
2	I10-I15	Hypertension	5231	9.2
3	E10-E14	Diabetes mellitus	4467	7.8
4	I60-I69	Cerebrovascular diseases	4289	7.5
5	U07-U09	COVID-19	3843	6.7
6	C33-C34	Malignant neoplasms of the lung	2879	5.1
7	C18-C21	Malignant neoplasms of the colon	2056	3.6
8	I70	Atherosclerosis	1836	3.2
9	J40-J47	Bronchitis, emphysema and asthma	1616	2.8
10	K70; K73-K74	Chronic liver disease and cirrhosis	1002	1.8
Total 10 causes			34,144	59.9
Total deaths			56,979	

**Table 3:** A ten-point plan for cardio-kidney-metabolic health in Croatia.

1	Early detection of elevated BP and elevated LDL cholesterol	- Introduce regular BP and lipid panel checks in adults at least once a year at the family practice. - Include data in the single CKM panel of the central healthcare information system of the Republic of Croatia (CEZIH).
2	Changes in poor dietary habits with an emphasis on reducing salt intake, increasing potassium intake, and fostering physical activity	- Change school and university curricula by increasing the number of physical education classes, allowing for physical activity in the workplace to change sedentary lifestyles. - Change of menus in kindergartens, schools, students and workers’ canteens and restaurants (reducing salt, use of salt substitutes, increasing vegetables, eliminating trans fatty acids, increasing the use of olive oil, fish etc.). Actual implementation requires the provision of appropriate foodstuffs and additional training of cooks to be able to follow the new recipes. - Continue, expand and regulate cooperation with the food industry. - Encourage the use of salt substitute (KCl) in primary and secondary prevention with required caution in patients with advanced stage CKD, and those taking potassium-sparing medications.
3	Reduce the number of smokers	- Provide education on the harmfulness of smoking. - Prohibit smoking in public areas including cafés. - Prohibit and provide strict sanctions for selling any form of tobacco (and nicotine) products to children (this pertains to new products—e-cigarettes, heated tobacco and so-called snuff). - Provide a larger number of places that provide help to those who want to quit smoking, so called schools of non-smoking.Raise awareness on how, for new products (e-cigarettes and heated tobacco), although containing fewer toxic ingredients, there is no evidence of a beneficial effect on CV health, and should be used only in smokers who are trying to quit smoking.
4	Regular physical examinations in adults over the age of 30	- Enable all citizens to have regular physical examinations which must become mandatory. - Panel tests on those examinations, in addition to medical history, basic clinical examination and proper BP measurement, should contain basic elements necessary to properly assess CKM health (urine, glucose, lipid panel (determination of lp (a) once in a lifetime), serum creatinine and estimated glomerular filtration, albumin-creatinine ratio for persons who are already at moderate CV risk, assessment of overall CV risk and CKD stage, and ECG.
5	Screening for familial hypercholesterolemia	- Launch a national plan and make it mandatory for all children when enrolling in primary school.
6	Prepare and publish clear protocols for diagnosis, treatment and monitoring of patients with a history of CV events	- The protocols must be concise, feasible and practical to provide for efficient clinical work, while also educational to increase the health literacy of medical doctors and thus reduce clinical inertia. - The protocols must specify the role of other healthcare professionals, e.g., pharmacists, and active role of the patients.
7	Define and regularly monitor patients with high CV risk who have not yet experienced a CKM event	- These patients in particular should have physical examinations as specified above once a year or more frequently as per the physicians’ assessment, i.e., prepared protocols. - Those patients should have their overall CKM health and total risk assessed. - Those patients should be additionally educated to change their poor habits actively and permanently, and to understand the importance of regular medication.
8	Increase access to diagnostics and patient monitoring through day hospitals	- There are multiple advantages to treatment and monitoring in day hospitals, because in addition to the possibility of monitoring and treating a larger number of patients, great financial savings can also be achieved. - It is necessary to educate physicians additionally on mutual communication, since it is practically a rule that patients are followed by several specialists (family medicine, cardiology, neurology, hypertensiology, etc.).
9	Increase the number of outpatient centers for CV rehabilitation	- The current number of such centers is insufficient.
10	Monitoring of treatment outcomes	- Digitalization aimed at connecting hospital systems to CEZIH for healthcare professionals providing care to the same patient to have access to all data (family practitioners, hospital specialists, pharmacists). - Setting up and connecting CKM patient registers. - Fast and successful integration into the European Health Data Space that will make it possible for our healthcare professionals, researchers and institutions to access health data from the entire European Union and, thus, enable better policymaking and the development of new health technologies.

## Data Availability

Not applicable.
